# Respiratory distress syndrome: influence of management on the hemodynamic status of ≤ 32-week preterm infants in the first 24 hours of life

**DOI:** 10.5935/0103-507X.20190056

**Published:** 2019

**Authors:** Daniela Matos Fiorenzano, Gabriela Nunes Leal, Karen Saori Shiraishi Sawamura, Alessandro Cavalcanti Lianza, Werther Brunow de Carvalho, Vera Lúcia Jornada Krebs

**Affiliations:** 1 Disciplina de Neonatologia, Departamento de Pediatria, Instituto da Criança, Hospital das Clínicas, Faculdade de Medicina, Universidade de São Paulo - São Paulo (SP), Brasil.; 2 Serviço de Ecocardiografia Neonatal e Pediátrica, Instituto da Criança, Hospital das Clínicas, Faculdade de Medicina, Universidade de São Paulo - São Paulo (SP), Brasil.

**Keywords:** Infant premature, Pulmonary surfactants, Respiratory distress syndrome, newborn, Hemodynamics, Echocardiography

## Abstract

**Objective:**

To investigate the influence of respiratory distress syndrome management on clinical and echocardiographic parameters used for hemodynamic evaluation in ≤ 32- week newborns.

**Methods:**

Thirty-three ≤ 32-week newborns were prospectively evaluated and subjected to invasive mechanical ventilation. The need for exogenous surfactant and clinical and echocardiographic parameters in the first 24 hours of life was detailed in this group of patients.

**Results:**

The mean airway pressure was significantly higher in newborn infants who required inotropes [10.8 (8.8 - 23) cmH_2_O *versus* 9 (6.2 - 12) cmH_2_O; p = 0.04]. A negative correlation was found between the mean airway pressure and velocity-time integral of the pulmonary artery (r = -0.39; p = 0.026), right ventricular output (r = -0.43; p = 0.017) and measurements of the tricuspid annular plane excursion (r = -0.37; p = 0.036). A negative correlation was found between the number of doses of exogenous surfactant and the right ventricular output (r = -0.39; p = 0.028) and pulmonary artery velocity-time integral (r = -0.35; p = 0.043).

**Conclusion:**

In ≤ 32-week newborns under invasive mechanical ventilation, increases in the mean airway pressure and number of surfactant doses are correlated with the worsening of early cardiac function. Therefore, more aggressive management of respiratory distress syndrome may contribute to the hemodynamic instability of these patients.

## INTRODUCTION

The immediate postnatal period is marked by important cardiopulmonary changes. In preterm infants with a gestational age of less than 32 weeks, the immaturity of several organs may affect this physiological transition, and a need for respiratory and hemodynamic support is common.^(1)^

Infant respiratory distress syndrome (RDS) is one of the main causes of morbidity in these patients. Surfactant deficiency, which is characteristic of developing lungs, causes alveolar collapse, which manifests as respiratory discomfort during the first hours of life.^(2)^ Treatment consists of pulmonary recruitment by applying positive airway pressure through noninvasive or invasive ventilation associated or not associated with the use of exogenous surfactant.^(3)^

The use of positive airway pressure facilitates the establishment of a functional residual capacity, decreases pulmonary vascular resistance and may increase pulmonary venous return, thereby favoring systemic output.^(4)^ In turn, the excessive use of positive airway pressure causes adverse effects, such as increased pulmonary vascular resistance, reduced pulmonary perfusion^(5)^ and venous return and a low cardiac output.^(6)^

A low systemic output and decreased superior vena cava (SVC) flow are associated with an increased risk of intraventricular hemorrhage in preterm infants;^(7)^ therefore, gaining a better understanding of the interaction between respiratory support and cardiac function is essential. Additionally, establishing whether the echocardiographic changes are transient and typical of perinatal cardiorespiratory adaptation or secondary to respiratory management of the patients is important.

The objective of the present study was to evaluate the influence of RDS management, comprising ventilatory support and pulmonary surfactant replacement, on clinical and echocardiographic parameters used for hemodynamic evaluation of ≤ 32-week newborns in their first 24 hours of life.

## METHODS

A prospective cross-sectional study was performed on a convenience sample of preterm infants admitted to the Neonatal Center of the *Instituto da Criança* of the *Hospital das Clínicas* of *Faculdade de Medicina* of the *Universidade de São Paulo* between August 2016 and March 2018. The Research Ethics Committee of the institution approved the research project (CAPPesq report: 1531501), and informed consent forms were signed by the legal guardians.

Newborn infants born with a gestational age ≤ 32 weeks who underwent invasive mechanical ventilation for RDS treatment within their first 24 hours of life were included. The gestational age was determined by obstetric ultrasound performed up to the 20^th^ week of gestation. Patients with congenital malformations, newborn infants who did not have an echocardiogram within the first 24 hours of life and those whose caregivers did not consent to the study were excluded.

The following clinical parameters were investigated: exposure to antenatal corticosteroids; presence of fetal distress; weight at birth; gestational age; sex; Score for Neonatal Acute Physiology Perinatal Extension, version II (SNAPPE II);^(8)^ greater mean airway pressure (Paw) in cmH_2_O during the first 24 hours of life; need for administration of exogenous surfactant and the number of doses (poractant alfa, 100mg/kg/dose); presence of hypotension (defined as the mean arterial pressure - MAP - less than the gestational age or a MAP lower than 30mmHg);^(9)^ and use of inotropic agents.

An echocardiogram was performed in the first 24 hours of life. This period was chosen because it is considered to have the highest risk for the occurrence of low output related to the transitional phase of circulation in the preterm newborn.^(10)^ A Logiq-e^®^ (GE Health Care^®^) portable ultrasound with a multifrequency transducer (6S) from 5 to 7.5MHz was used for morphological and functional cardiac evaluation by a team composed of three experienced echocardiographers who were blinded to the patient's clinical parameters. The cavity measurements and cardiac function evaluation were performed according to the 2010 guidelines of the American Society of Echocardiography (ASE).^(11)^

The diastolic and systolic diameters of the left ventricle were measured in M mode, which allowed calculation of the fractional shortening (ΔD) and left ventricular ejection fraction (LVEF) using the Teichholz method.^(11)^ Measurements of the tricuspid annular plane systolic excursion (TAPSE)^(12)^ and mitral annular plane systolic excursion (MAPSE)^(13)^ allowed calculation of the z-scores of these parameters for each patient.

The velocity-time integrals for the pulmonary artery flow (VTIPA) and SVC were obtained using Doppler. To estimate the VTIPA, the Doppler sample was placed in the right ventricular outflow tract (RVOT) in the parasternal short axis view just below the pulmonary valve plane. The VTI of the VSC was estimated by placing the Doppler sample at the entrance of the vena cava in the right atrium in the subcostal section view. The maximum diameter of the RVOT was measured using a two-dimensional, parasternal, short axis view. In contrast, the mean SVC diameter was recorded as mode M in the parasternal view of the RVOT.

The right ventricular output (RVO) was calculated according to the following formula:

RVO=heartrate×VTIPA×π×diameteroftheoutflowtract2÷4×weight

With the following units: VTI in cm; outlet diameter in cm; weight in kg; and π = 3.14.

The SVC flow (SVCF) was calculated using the following formula:

SVCF:heartrate×VTI×π×meandiameterofSVC2÷4×weight

With the following units: VTI in cm; diameter of the outflow tract in cm; weight in kg; and π = 3.14.

The cut-off points used to categorize the echocardiographic parameters of cardiac function as normal (no dysfunction) or reduced (with dysfunction) were ΔD 28%,^(14)^ LVEF 55%,^(14)^ RVO 150mL/kg/minute,^(15)^ pulmonary VTI 7cm,^(16)^ MAPSE z-score < -2,^(17)^ TAPSE z-score < -2^(18)^ and SVCF 40mL/kg/minute.^(15)^

The RVO was used to avoid overestimating the systemic output given the likelihood of a patent ductus arteriosus on the first day of life with a *shunt* from left to right.^(19)^

In the statistical analysis, the qualitative variables were described as absolute frequencies and percentages. Quantitative variables were described using summary statistics (mean, standard deviation - SD) and the median, minimum and maximum.

To assess the correlation between the MAP and Paw and the echocardiographic measurements, Spearman's correlation coefficient (r) was used. The comparison of Paw between the groups with and without dysfunction was performed using the nonparametric Mann-Whitney test. The association between the number of doses of exogenous surfactant and the presence of cardiac dysfunction was assessed using the Kruskal-Wallis test. The significance level was set as p < 0.05. The data analysis was performed using the IBM Statistical Package for Social Sciences for Windows, version 20.0.

## RESULTS

A total of 33 preterm infants on mechanical ventilation for treatment of RDS were studied, including 63.6% males and 36.4% females. The means (± SDs) for gestational age, weight and SNAPPE II were 27.8 (± 1.9) weeks, 958 (± 332) grams and 39.5 (± 20.6), respectively. Fetal distress occurred in 33.3% of the cases, and 78.8% of the fetuses were exposed to antenatal corticosteroids.

The median of the mean Paw was 9.2cmH_2_O (minimum of 6.2 and maximum of 23cmH_2_O). Four patients were treated with high-frequency ventilation by interrupting flow. A total of 32 patients were given at least one dose of exogenous surfactant.

Hypotension was diagnosed in 11 (33%) patients; a total of 8 (24%) patients were given inotropes (dobutamine, adrenaline or dopamine) in the first 24 hours of life. No significant correlation was found between the MAP and mean Paw value (r = -0.030; p = 0.869). The mean Paw was higher in those that required inotropes: 10.8 (8.8 - 23) cmH_2_O *versus* 9 (6.2 - 12) cmH_2_O, with p = 0.04.

The echocardiogram was performed within a median of 20 hours of life (minimum of 7 and maximum of 24 hours). No significant association was detected between the echocardiographic measurements and fetal distress or use of antenatal corticosteroids. Additionally, no correlation was found between the MAP and echocardiographic parameters of cardiac function (Table 1). A negative correlation was found between the mean Paw and the VTIPA, RVO and TAPSE (Table 1). A positive correlation was found between the VTIPA and RVO (r = 0.796, p < 0.0001).

**Table 1:** Correlation between the mean arterial and mean airway pressures and echocardiographic parameters

	MAP (mmHg)			Mean Paw (cmH_2_O)		
	n	r	p value	n	r	p value
ΔD	31	0.007	0.968	32	-0.238	0.190
LVEF	32	0.059	-0.157	33	-0.234	0.191
MAPSE	31	-0.157	0.398	32	-0.262	0.147
VTIPA	32	-0.023	0.900	33	-0.388*	0.026
RVO	30	0.100	0.598	31	-0.427*	0.017
TAPSE	31	-0.254	0.169	32	-0.372*	0.036
SVCF	31	0.215	0.245	32	-0.085	0.642
DD	32	-0.109	0.553	33	0.004	0.982

ΔD - left ventricle shortening fraction; LVEF - left ventricular ejection fraction; MAPSE - mitral annular plane systolic excursion; VTIPA - velocity-time integral of the pulmonary artery; RVO - right ventricular output; TAPSE - tricuspid annular plane systolic excursion; SVCF - superior vena cava flow; DD - duct diameter; MAP - mean arterial pressure; Mean Paw - mean airway pressure; r = Spearman correlation coefficient.

* Statistical significance.

Table 2 shows the frequency of cardiac dysfunction according to the echocardiographic criteria.

**Table 2:** Frequency of cardiac dysfunction according to the echocardiographic criteria studied

Frequency	n (%)
ΔD < 28%	5 (15.2)
LVEF < 55%	5 (15.2)
MAPSE z-score < -2	3 (9.4)
VTIPA < 7cm	17 (51.5)
RV < 150mL/kg/minute	8 (24.2)
TAPSE z-score < -2	2 (6.3)
SVCF < 40mL/kg/minute	5 (15.2)

ΔD - left ventricle shortening fraction; LVEF - left ventricular ejection fraction; MAPSE - mitral annular plane systolic excursion; VTIPA - velocity-time integral of the pulmonary artery; RV - right ventricle; TAPSE - tricuspid annular plane systolic excursion; SVCF -  superior vena cava flow.

The mean Paw values were higher in patients with cardiac dysfunction based on the following parameters: ΔD < 28%, LVEF < 55%, VTIPA < 7cm and RV < 150mL/kg/minute (Table 3).

**Table 3:** Mean airway pressure values according to echocardiographic parameters of cardiac dysfunction

Echocardiography	n	Median mean Paw (cmH_2_O) (minimum - maximum)	p value*
ΔD < 28%			
Yes	5	11.0 (10.4 - 23.0)	0.020
Not applicable	27	9.0 (6.2 - 12.0)	
LVEF < 55%			
Yes	5	11.0 (10.4 - 23.0)	0.020
Not applicable	28	9.0 (6.2 - 12.0)	
MAPSE z-score < -2			
Yes	3	10.7 (10.0 - 13.4)	0.045
Not applicable	29	9.1 (6.2 - 23.0)	
VTIPA < 7cm			
Yes	17	9.6 (8.2 - 13.4)	0.035
Not applicable	16	8.8 (6.2 - 23.0)	
RV < 150mL/kg/minute			
Yes	8	10.5 (8.8 - 13.4)	0.020
Not applicable	24	9.0 (6.2 - 23.0)	
TAPSE z-score < -2			
Yes	2	9.2 (7.7 - 23.0)	0.086
Not applicable	30	9.0 (7.4 - 12.0)	
SVCF < 40mL/kg/minute			
Yes	5	12.0 (8.2 - 23.0)	0.132
Not applicable	27	9.1 (6.2 - 12.0)	

Mean Paw - mean airway pressure; ΔD - left ventricle shortening fraction; LVEF - left ventricular ejection fraction; MAPSE - mitral annular plane systolic excursion; VTIPA - velocity-time integral pulmonary artery; RV - right ventricle; TAPSE - tricuspid annular plane systolic excursion; SVCF - superior vena cava flow.

* Nonparametric Mann-Whitney test.

A negative correlation was noted between the number of exogenous surfactant doses and the RVO (r = -0.394; p = 0.028) and VTIPA (r = -0.355; p = 0.043) measurements. A higher number of surfactant doses was used for patients with cardiac dysfunction based on the following echocardiographic parameters: VTIPA < 7cm, RV < 150mL/kg/minute and TAPSE z-score < -2 (Table 4).

**Table 4:** Association between the number of surfactant doses and the presence of ventricular dysfunction

Echocardiography	Surfactant doses			p value*
	0 n (%)	1 n (%)	≥ 2 n (%)	
ΔD < 28%	0 (0.0)	3 (20.0)	2 (12.5)	0.708
LVEF < 55%	0 (0.0)	3 (18.8)	2 (12.5)	0.999
MAPSE z-score < -2	0 (0.0)	1 (6.7)	2 (12.5)	0.999
VTIPA < 7cm	0 (0.0)	5 (31.3)	12 (75.0)	0.022
RV < 150mL/kg/minute	0 (0.0)	1 (6.7)	7 (43.8)	0.049
TAPSE z-score < -2	0 (0.0)	1 (6.7)	1 (6.3)	0.999
SVCF < 40mL/kg/minute	0 (0.0)	3 (20)	2 (12.5)	0.708

ΔD - left ventricle shortening fraction; LVEF - left ventricular ejection fraction; MAPSE - mitral annular plane systolic excursion; VTIPA - velocity-time integral of the pulmonary artery; RV - right ventricle; TAPSE - tricuspid annular plane systolic excursion; SVCF - superior vena cava flow.

* Nonparametric Kruskal-Wallis test.

## DISCUSSION

The present study demonstrated the impact of respiratory management on the hemodynamic status of ≤ 32-week newborn infants using not only clinical parameters (MAP and need for inotropic drugs) but also bedside echocardiographic parameters of cardiac function.

Increases in the mean Paw indicated worsening of right and left cardiac functions as evaluated by echocardiography and a greater need for hemodynamic support with inotropic drugs.

Other authors have described a worsening of myocardial function in patients with moderate to severe respiratory failure. In 1996, Evans and Kluckow showed a reduction in the output of both ventricles that was associated with an increased severity of respiratory failure and the frequency of low output (< 150mL/kg/minute) of the LV and RV with the increase in the mean Paw.^(20)^ In a more recent study, de Waal et al. demonstrated a reduction of the RVO after an increase in the positive end-expiratory pressure (PEEP) but not of the systemic output estimated by the SVCF.^(21)^

Although the ΔD and LVEF measurements are less reliable in neonates (due to the paradoxical movement of the interventricular septum secondary to high pressures in the RV),^(22)^ we were able to demonstrate an association between the reduction of these left cardiac function parameters and an increase in the mean Paw in our sample.

In the present study, the negative correlations between the mean Paw and the VTIPA, RVO and TAPSE measurements and the higher frequencies of a VTIPA < 7cm and RV < 150mL/kg/minute with the increase in the mean Paw reinforce the influence of the latter parameters on the RV. Since a strong correlation exists between the VTIPA and RVO and the former is simpler to measure because it does not require calculation, measuring this parameter to quickly estimate the systolic function of the VD may be an interesting approach.

The effect of the mean Paw on the RVO is most likely due to reduced venous return.^(20,21)^ However, hypoxemia secondary to respiratory failure may have a direct effect on the increase in pulmonary vascular resistance^(5)^ and can lead to some degree of subendocardial ischemia, which causes impairment of myocardial function.^(23)^

The finding of the inverse association between the number of surfactant doses and the presence of a VTIPA < 7cm and RV < 150mL/kg/minute differed from observations in the literature. Sehgal et al. reported an increase in the RVO and a reduction in the LV output after surfactant was given in the first hour of life.^(24)^ Additionally, Vitali et al. did not observe changes in ventricular function measurements 2 hours after surfactant administration but described an improvement in the output of both ventricles and the TAPSE 24 hours later.^(25)^ In fact, the administration of exogenous surfactant is followed by pulmonary recruitment, which results in a reduction of pulmonary vascular resistance.^(5)^ Therefore, the following outcomes would be expected: an increase in the right ventricular output due to a reduced afterload and possibly a decrease in systemic perfusion due to an increase in left-to-right shunting through the ductus arteriosus, which usually is patent on the first day of life in preterm infants.^(24)^

In the present study, the worst cardiac function was exhibited by patients who used a greater number of surfactant doses and could be explained, in part, by two factors. First, we can assume that newborn infants requiring two or more doses of surfactant have more severe respiratory failure; second, in our sample, all patients who received more than one dose of surfactant were on mechanical ventilation at the time of the echocardiogram. Therefore, the hemodynamic repercussions of mechanical ventilation may have prevailed over the effects of exogenous surfactant on pulmonary compliance and vascular resistance.

The study has some limitations, such as the relatively small number of newborns. In addition, the type of ventilation used (four patients received high-frequency flow interruption ventilation) and the mean Paw may have favored the presence of cardiac dysfunction.^(26)^

The fact that the patients were evaluated at only one time point (within the first 24 hours of life) should also be emphasized. During this period, preterm infants are prone to myocardial dysfunction.

## CONCLUSION

In newborn infants with a gestational age ≤ 32 weeks under invasive mechanical ventilation, more aggressive management of respiratory distress syndrome seems to exert a negative effect on echocardiographic parameters of cardiac function, particularly the right ventricle. New multicenter studies with a larger number of patients should be conducted to improve treatment strategies for this group of patients.
